# A Rare Case of Unprovoked Deep Cerebral Venous Sinus Thrombosis With Associated Petechial Hemorrhage

**DOI:** 10.7759/cureus.49442

**Published:** 2023-11-26

**Authors:** Abhinav K Rao, Ian Whitaker, Theshali Anthony, Nick Shaheen, Aidan Lum Kong

**Affiliations:** 1 Internal Medicine, Trident Medical Center, North Charleston, USA; 2 Radiology, Aultman Hospital, Canton, USA; 3 Interventional Radiology, Northeast Ohio Medical University, Rootstown, USA

**Keywords:** cerebral venous and dural sinus thrombosis, therapeutic anticoagulation, hemorrhage, dural venous thrombosis, seizure, cerebral venous thrombosis

## Abstract

A 73-year-old male with hyperlipidemia and prior squamous cell carcinoma presented with a new-onset generalized tonic-clonic seizure and left-sided weakness. He had a progressively worsening cervical and occipital headache for nine days and was initially evaluated with an unremarkable CT head. The patient arrived at the emergency department with a left gaze deviation, prompting a "Code Neuro." CT angiography (CTA) detected a large dural sinus thrombosis, which was confirmed with a CT venogram and brain MRI. The patient was closely monitored in the intensive care unit and managed with heparin, nicardipine, and hypertonic saline boluses. Neurosurgery opted against surgical intervention. EEG revealed right hemisphere focal dysfunction. Recanalization occurred on hospital day 6. The patient was transitioned to apixaban upon discharge and instructed to follow up with hematology for a hypercoagulable workup.

This case highlights the diagnostic complexity of cerebral venous sinus thrombosis (CVST), urging consideration of a differential even in atypical patients. We hope reporting this case will prompt clinicians to consider CVST in patients with non-specific symptoms, leading to timely diagnosis and treatment.

## Introduction

Headaches are a common complaint encountered by internists and have an extensive differential often requiring the exclusion of life-threatening conditions. Cerebral venous sinus thrombosis (CVST), despite its rarity with an estimated annual incidence of 1.16 to 2.02 per 100,000 people, can also present with headaches. Notably, headaches manifest in 90% of CVST cases, and the condition primarily affects females with an average age of diagnosis of 37 years [1-2]. There are several known genetic and acquired risk factors, and the gender imbalance is likely connected to pregnancy, puerperium, and the use of oral contraceptives.

Notably, up to 30% of patients have a normal head CT [3]. Diagnosis thus relies on abnormal brain MRI findings in venous sinuses confirmed by a lack of flow on magnetic resonance venography (MRV). Thrombophilia screening is indicated for patients with a personal and/or family history of venous thrombosis, CVST at a young age, or in the absence of an identifiable risk factor. Fortunately, CVST has a favorable prognosis with nearly 80% of patients achieving complete recovery at 16 months [4].

## Case presentation

The patient is a 73-year-old Caucasian male with a past medical history of hyperlipidemia and remote cutaneous squamous cell carcinoma of the nose post excision who presented after a new-onset generalized tonic-clonic seizure with residual left-sided weakness. The patient developed a progressively worsening headache nine days prior to presentation localized to the cervical and occipital regions and was seen in urgent care where his CT head was unremarkable. He denied other complaints at that time. His family history was significant for colon cancer in the father and breast cancer in the mother. There was no significant personal or family history of hypercoagulable disorders.

**Figure 1:**
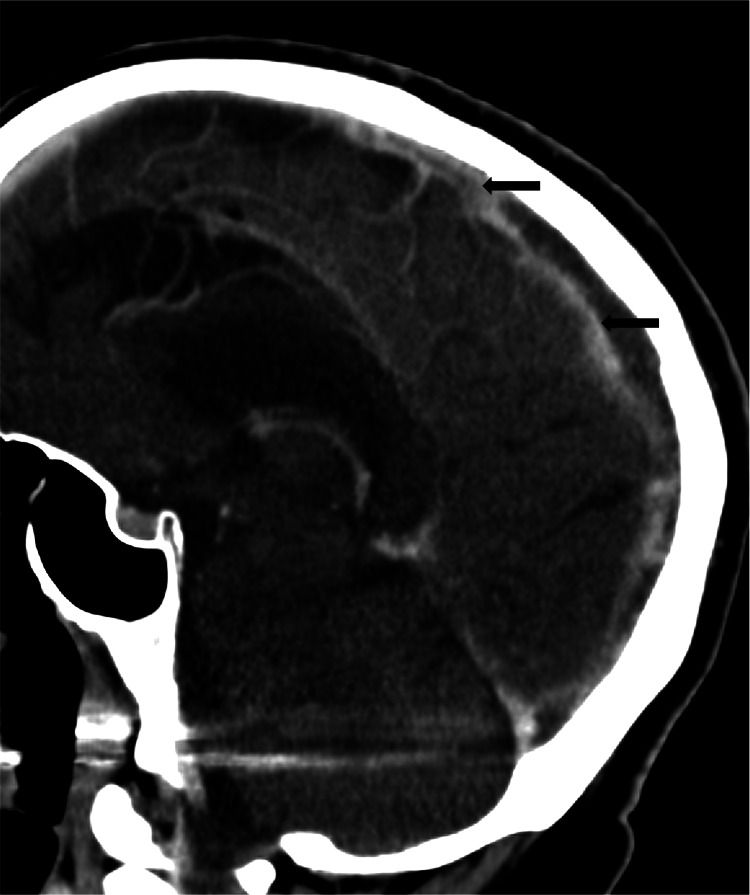
Sagittal CTV demonstrates subtotal occlusive thrombus in the superior sagittal sinus and torcular Herophili

**Figure 2:**
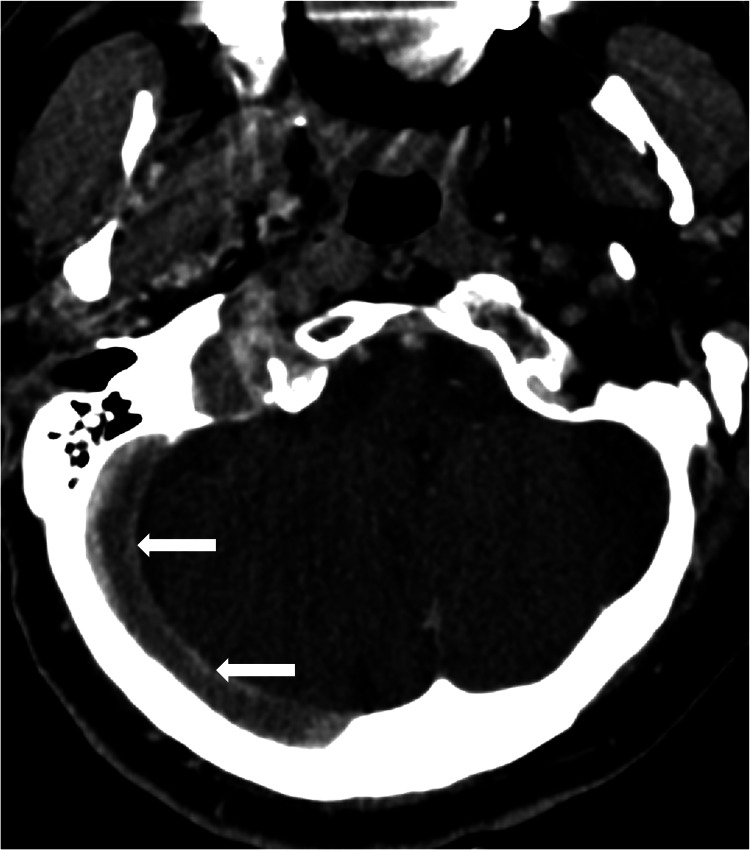
Axial CTV demonstrates subtotal occlusive thrombus filling the right transverse sinus, sigmoid sinus, and jugular bulb Not shown is the partial extension into the right internal jugular vein.

**Figure 3:**
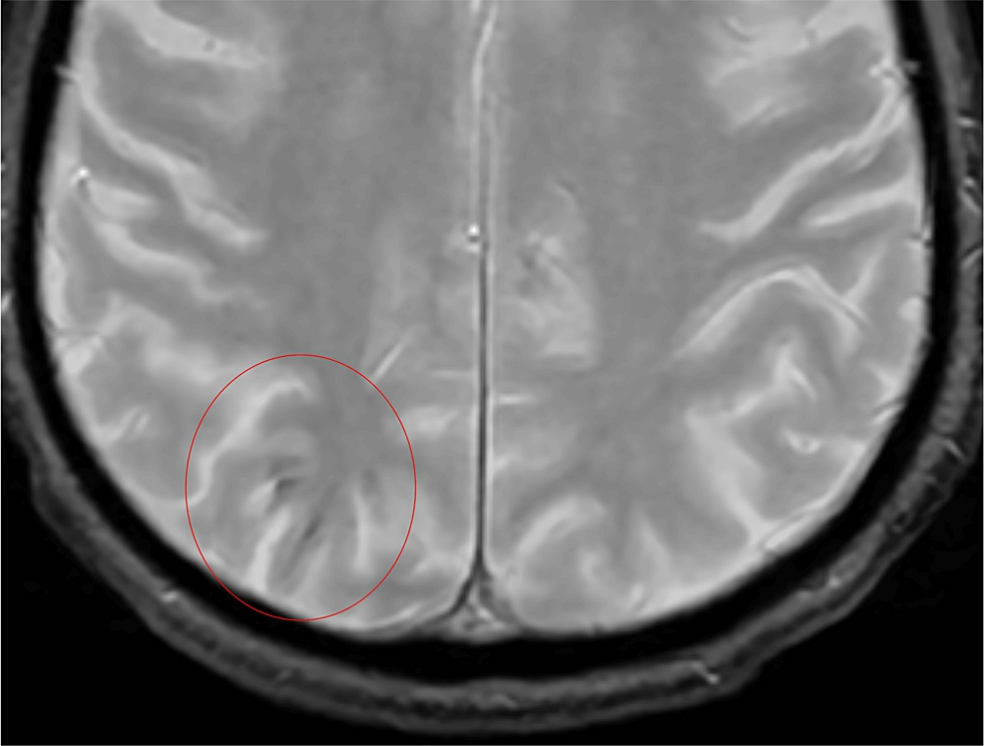
Gradient echo axial sequence demonstrates subtle blooming in the right parietal lobe compatible with petechial hemorrhage

Upon emergency department arrival, the patient was post-ictal with a left gaze deviation. "Code Neuro" was activated. CT head ruled out intracranial hemorrhage, while CTA revealed filling defects in the superior sagittal and right transverse sinuses extending into the right internal jugular vein suggesting dural sinus thrombosis. Subsequent CT venogram (CTV) confirmed extensive thrombosis in these sinuses and the right internal jugular vein, along with ill-defined foci. A subsequent brain MRI indicated those foci likely resulted from engorged or thrombosed cortical veins with minor associated petechial hemorrhage (Figures 1-3). Another witnessed seizure in the emergency department was managed with lorazepam, and a loading dose of levetiracetam was initiated.

The patient was admitted to the intensive care unit with a heparin drip with a target partial thromboplastin time of 60-80. A nicardipine drip was initiated for strict blood pressure control to maintain systolic blood pressure between 140 and 160 mmHg. Normal saline at 125 cc/hour was administered to maintain adequate volume status with a sodium goal of 145-50 using 3% hypertonic boluses. Neurosurgery did not recommend surgical intervention or external ventricular drain placement after evaluating the patient. EEG revealed focal dysfunction in the right hemisphere likely due to a known structural cause, with no epileptiform waveforms.

**Figure 4:**
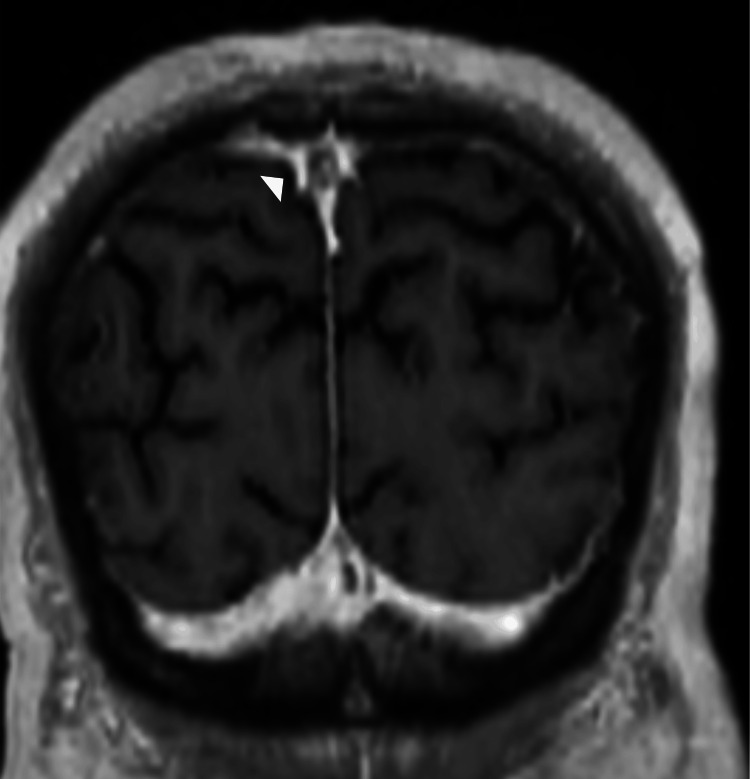
Three-month follow-up MRI coronal volumetric T1 postcontrast sequence demonstrates near complete resolution with minimal persistent thrombus in the superior sagittal sinus

The patient continued on heparin with close neurochecks. Thrombosis persisted on serial imaging until hospital day 6 when a repeat CTA revealed recanalization in the right transverse and sigmoid sinuses with improved flow. A perfusion study indicated a small wedge-shaped infarct in the right occipital lobe due to dural venous sinus thrombosis. Throughout the hospital course, the patient was closely monitored by neurosurgery and neurology. At discharge, the patient was transitioned to apixaban with close outpatient follow-up with neurology and hematology. After three months of continuing apixaban outpatient, a follow-up MRI revealed a near-complete resolution of the thrombus (Figure 4).

## Discussion

CVST is a rare diagnosis and has an annual incidence of 1.16 to 2.02 per 100,000 primarily affecting females. The average age of diagnosis is approximately 37 years [1]. Risk factors include infection, pregnancy and puerperium, obesity, dehydration, head injury, mechanical precipitants, drugs (i.e., oral contraceptives, hormone replacement therapy), inflammatory diseases, malignancy, genetic or acquired prothrombotic state, and central nervous system disorders.

The most frequently reported symptom of CVST is headaches occurring in almost 90% of patients [2]. Notably, up to 30% of patients have normal head CTs [3]. Diagnosis relies on abnormal brain MRI findings in venous sinuses confirmed by a lack of flow on the MRV. Thrombophilia screening is indicated for patients with a personal and/or family history of venous thrombosis, CVST at a young age, or in the absence of an identifiable risk factor.

Fortunately, CVST has a favorable prognosis. In the International Study of Cerebral Vein and Dural Venous Thrombosis (624 patients), the primary cause of early CVST-related deaths was transtentorial herniation secondary to a large hemorrhagic lesion. Notably, 79% of patients achieved complete recovery at 16 months [4].

This case details an unusual case of CVST in an elderly White male without any identified risk factors. Numerous risk factors have been associated with CVST, with more than 85% of patients having at least one identified risk factor [5]. Generally, these risk factors involve conditions that promote a prothrombotic state. Genetic and acquired thrombophilia collectively account for approximately 34% of CVST cases. While a history of squamous cell carcinoma could certainly be considered a prothrombotic state, studies have shown most cancer-related CVST develops within four months of cancer diagnosis which was not the case in our patient [6]. This lack of risk factors underscores the importance of maintaining a broad differential diagnosis and performing appropriate imaging to prevent missed diagnosis. For this patient, a hypercoagulable workup is indicated once the patient has been stabilized to evaluate for underlying thrombophilia. This patient ultimately had a negative thrombophilia workup.

The mainstay of treatment for CVST remains early anticoagulation with either heparin or low-molecular-weight heparin at therapeutic doses. One study demonstrated that more than 80% of CVST patients were treated with anticoagulants indicating consensus among neurologists on the effectiveness of anticoagulation in CVST [7]. Other management strategies include thrombolysis or direct thrombectomy. As clinicians become more skilled in these approaches, further research is warranted to select the populations that will most benefit from these more aggressive interventions. Studies so far have identified several risk factors that carry an increased risk of an unfavorable outcome. The risk factors include male sex, age above 37 years, presenting with coma, comorbid mental status disorder, intracranial hemorrhage on admission, thrombosis of the deep cerebral venous system, central nervous system infection, and known cancer diagnosis [8]. At this time, it is not established if these higher-risk patients would benefit from more aggressive interventions in the management of CVST.

## Conclusions

This report demonstrates the diagnostic challenges associated with CVST, particularly in an atypical patient. The initial symptoms of CVST are non-specific and can mimic other conditions including stroke or complex migraine. Given the potential severity of CVST, even though it is rare, it should remain a differential diagnosis until ruled out with appropriate imaging modalities such as MRV or, alternatively, CTV. While diagnosing CVST can be challenging, early intervention with anticoagulation, thrombolysis, or surgical approaches can improve patient outcomes. Further research is warranted to direct management strategies for patients who do not fit into the typical demographic profile associated with CVST and who have a higher risk of unfavorable outcomes.
